# Aggressive and Metastatic Pituitary Neuroendocrine Tumors: Therapeutic Management and Off-Label Drug Use

**DOI:** 10.3390/jcm13010116

**Published:** 2023-12-25

**Authors:** Pedro Iglesias

**Affiliations:** Department of Endocrinology, Hospital Universitario Puerta de Hierro Majadahonda, Instituto de Investigación Sanitaria Puerta de Hierro Segovia de Arana (IDIPHISA), 28222 Madrid, Spain; piglo65@gmail.com

**Keywords:** pituitary neuroendocrine tumors, surgery, radiotherapy, temozolomide, dopamine agonists, somatostatin analogs, radionuclide treatment, immunotherapy

## Abstract

Pituitary neuroendocrine tumors (PitNETs) are the most common pituitary tumors and the second most common brain tumors. Although the vast majority (>90%) are benign, a small percentage (<2%) are aggressive. These aggressive PitNETs (AgPitNETs) are defined by the presence of radiological invasion, a high rate of cell proliferation, resistance to conventional treatments, and/or a high propensity for recurrence. Lastly, there are the rare pituitary carcinomas, also known as metastatic PitNETs (MetPitNETs), which account for only 0.2% of cases and are defined by the presence of craniospinal or distant metastases. At present, there are no definitive factors that allow us to predict with certainty the aggressive behavior of PitNETs, making the therapeutic management of AgPitNETs a real challenge. Surgery is considered the first-line treatment for AgPitNETs and MetPitNETs. Radiation therapy can be effective in controlling tumor growth and regulating hormone hypersecretion. Currently, there are no approved non-endocrine medical therapies for the management of AgPitNETs/MetPitNETs, mainly due to the lack of randomized controlled clinical trials. As a result, many of the medical therapies used are off-label drugs, and several are under investigation. Temozolomide (TMZ) is now recognized as the primary medical treatment following the failure of standard therapy (medical treatment, surgery, and radiotherapy) in AgPitNETs/MetPitNETs due to its ability to improve overall and progression-free survival rates in responding patients over 5 years. Other therapeutic options include pituitary-targeted therapies (dopamine agonists and somatostatin analogs), hormonal antisecretory drugs, non-hormonal targeted therapies, radionuclide treatments, and immunotherapy. However, the number of patients who have undergone these treatments is limited, and the results obtained to date have been inconsistent. As a result, it is imperative to expand the cohort of patients undergoing treatment to better determine the therapeutic efficacy and safety of these drugs for individuals with AgPitNETs/MetPitNETs.

## 1. Introduction

Pituitary neuroendocrine tumors (PitNETs) are the second most common brain tumor, accounting for approximately 15% of all intracranial neoplasms [1]. Most cases are benign tumors that respond adequately to standard treatment, regardless of size (micro- or macroadenomas). At the other extreme are pituitary carcinomas, also called metastatic PitNETs (MetPitNETs) (very rare, 0.2%) characterized by the presence of cerebrospinal and/or distant metastases [2]. Among them are aggressive pituitary neuroendocrine tumors (AgPitNETs), which have been defined as radiologically invasive tumors with a high cell proliferation rate and unusually rapid tumor growth, resistant to conventional standard medical treatment, with multiple local recurrences despite repeated surgery and radiotherapy [3].

The clinical course of AgPitNET/MetNETs is often severe and has a significant impact on patients’ quality of life and survival. Therapeutic management of these tumors is a real challenge for clinicians. To date, no universally effective treatment has been established for all patients with AgPitNET/MetPitNET. Due to their low prevalence, it is very difficult to conduct clinical trials with large numbers of patients to evaluate the efficacy and safety of different therapeutic options. In addition, these tumors are highly heterogeneous and may respond differently to different types of drugs. As a result, and given their poor prognosis, individualized treatment trials are often required to assess efficacy and safety. All of this requires an individualized evaluation of each patient by a multidisciplinary committee experienced in this type of pathology, in which the best diagnostic and therapeutic options are considered in each case, considering the possibility of carrying out a multimodal treatment, sometimes with off-label drugs, to control both hormonal hypersecretion, if present, and tumor growth.

## 2. Definition and Epidemiology of Aggressive PitNETs

In 2018, the European Society of Endocrinology (ESE) proposed a set of criteria to consider for the diagnosis of AgPitNET [3]. Currently, it seems appropriate to consider at least three of the five criteria listed in Table 1 to establish a diagnosis of AgPitNET [4].

**Figure 1:**
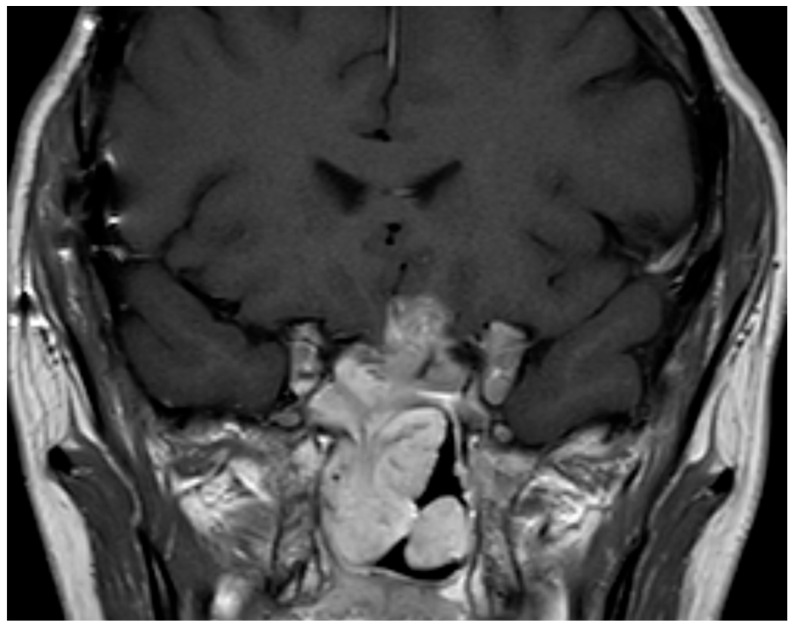
Coronal pituitary magnetic resonance image with T1-weighted contrast-enhanced T1 sequence showing the image of a persistent aggressive silent corticotropinoma after 4 surgeries plus radiotherapy.

One of the fundamental challenges in AgPitNETs is their early detection, which would provide the opportunity for more aggressive treatment soon after the initial diagnosis is confirmed. This diagnostic difficulty is due to the fact that the presence of an invasive tumor does not always indicate aggressive behavior and that there are no reliable histologic markers that indicate the presence of an AgPitNET. Although these tumors often exhibit markers of high cell proliferation, such as a Ki-67 index ≥3%, a high mitotic count (>2 mitoses per 10 high-power fields, HPFs), and positivity for p53 expression (>10 strongly positive nuclei per 10 HPFs), their presence also does not always predict aggressive behavior [3,5] (Figure 1).

According to the World Health Organization (WHO, 2022), the main histological subtypes of PitNETs that usually show aggressive behavior are shown in Table 2 [6,7].

The epidemiology of AgPitNETs is difficult to assess and the percentages vary widely depending on the definition used and the type of patients studied. This discrepancy is due to the lack of a definitive and universally accepted definition of AgPitNET, resulting in a lack of epidemiologic studies related to these tumors. Nevertheless, an aggressive clinical course is estimated to occur in approximately 0.5% of all PitNETs detected [8].

A recently published study conducted by the ESE Task Force on Aggressive Pituitary Tumours/Carcinomas using an electronic survey from August 2020 to May 2021 describes the clinical and pathological features and therapeutic outcomes in a large cohort (171 patients) of aggressive and metastatic pituitary tumors [9]. This study performed in 171 patients (mean age 46 years, range 3–64) showed a predilection for males (*n* = 107, 63%) and functioning tumors (*n* = 123, 71.9%). Among them, 70.8% (*n* = 121) were AgPitNETs and 29.2% (*n* = 50) MetPitNETs. The most common histological subtype was prolactinoma (*n* = 54, 31.6%), followed by corticotropinoma (*n* = 51, 29.8%) and non-functioning adenoma (*n* = 45, 26.3%). At diagnosis, 57% were considered benign tumors and 38% were considered aggressive. Regarding size, 74.4% were macroadenomas (≥1 cm), 22% were giant tumors (≥4 cm), and 3.5% were microadenomas (<1 cm). At the first surgery, the Ki67 index was ≥3% in 74/93 (80%) and ≥10% in 38/93 (41%) tumors. Mitotic count (*n* > 2) and p53 expression (≥10%) were found in 18/44 (40.9%) and 13/43 (30.2%) of the tumors, respectively. Finally, in Met PitNETs, metastases were diagnosed after a median of 6.3 years (IQR 3.7–12.1) from the initial diagnosis [9].

## 3. Therapeutic Management

An adequate therapeutic orientation to AgPitNET requires first of all the knowledge that we are dealing with this type of tumor. The lack of predictive factors for an aggressive clinical course makes the therapeutic management of these tumors a real challenge. Regardless of the radiologic studies of invasiveness, the results of the histologic study, and the markers of aggressiveness, the aggressive clinical behavior of the tumor is the most important factor in its diagnosis. In this sense, when a pituitary tumor that after conventional treatment (medical treatment, surgery, and radiotherapy), regardless of the initial response, behaves aggressively, either in the form of rapid growth of the tumor remnant or early tumor recurrence, it is necessary to consider new therapies, which can sometimes depend on the histology and functional capacity of the tumor.

The type of treatment for these tumors should be a multimodality approach. Surgical reintervention should be the first treatment after diagnosis. The possibility of re-irradiating the tumor should also be considered. Regarding medical treatment, chemotherapy, endocrine and non-endocrine targeted therapies, immunotherapy, and peptide receptor radionuclide therapy (PRRT) should be considered [2,4,9,10,11,12,13,14,15,16]. 

### 3.1. Surgical Reintervention (Debulking)

Surgery is considered the first-line therapy in AgPitNET recurrence, especially for the first recurrence. Approximately 80% of AgPitNETs require at least two surgeries, while approximately 30% require at least four surgeries [5]. The goals are to achieve the largest safe tumor resection possible, to decompress the optic pathways, to obtain new histopathologic samples of the tumor, and to minimize potential neurosurgical complications such as hypopituitarism, cerebrospinal fluid fistula, arginine vasopressin deficiency, and neurologic injury [5]. Complete macroscopic resection of these tumors is unlikely because they are large, invasive tumors with poorly defined borders involving multiple compartments. However, a reduction in tumor mass reduces the subsidiary tumor volume of radiotherapy and may also improve the response to various medical treatments. Suprasellar extensions can be managed through an extended endonasal endoscopic approach in tertiary care centers with experience. The transcranial approach should be globally reserved for tumors extending into the compartment lateral to the internal carotid artery [17,18,19,20]. Because of the surgical complexity of these tumors, it is recommended that surgery be performed at referral centers with teams of neurosurgeons with expertise in pituitary surgery [3,5,9,21,22,23].

### 3.2. Radiation Therapy 

Radiotherapy, when indicated, is part of the standard treatment for all pituitary tumors, and it achieves an excellent degree of tumor growth control [3]. It achieves 90–95% control of tumor growth at 5–10 years and 40–80% normalization of hormonal hypersecretion at 5 years in patients with prolactin-, GH-, and ACTH-secreting adenomas [24].

Radiotherapy is usually indicated in non-functioning PitNETS with an aggressive growth pattern with tumor persistence after surgery, whereas in functioning PitNETS both surgery and medical treatment prior to radiotherapy would be indicated. According to the ESE recommendations, adjuvant radiotherapy should be considered in the presence of an invasive tumor remnant with pathologic proliferation markers (Ki67 index, mitotic count, p53 immunodetection) indicating aggressive behavior [3].

In contrast to conventional radiotherapy, new techniques such as stereotactic radiosurgery (SR), fractionated stereotactic radiotherapy (FSTR), intensity-modulated radiotherapy (IMRT), image-guided radiotherapy (IGRT), and proton radiotherapy allow for the delivery of higher radiation doses to the target with rapid dose fall-off to surrounding normal tissues, potentially limiting the long-term toxicity of radiation [24,25,26,27,28,29]. FSTR is usually administered in 25–30 fractions, with a total dose ranging from 45–54 Gy. SR can be administered as a single dose of 12–14 Gy (maximum of 16 Gy). Fractionated SRS is recommended when a single dose may damage the optic pathway, in which case 25 Gy distributed in five fractions is usually administered [26]. Complications of radiation therapy include hypopituitarism, vascular damage, secondary malignancies, and optic nerve damage [24].

In the recent study by Burman et al. [9], 89% (152 of 171) of AgPitNET/MetPitNET patients received radiotherapy, with 36.2% (55 of 152) receiving two or more courses. The therapeutic response to the first course of radiotherapy according to the modified response evaluation criteria in solid tumors (RECIST) was a complete response (CR) in 3.2%, partial response (PR) in 41.9%, stable disease (SD) in 47.6%, and progressive disease (PD) in 7.3%. The median time between the first and second course of radiotherapy was 5.4 years (IQR: 3.5–8.9 years), and the therapeutic response rate was similar to the first course.

### 3.3. Off-Label Medical Therapies

Currently, there are no approved non-endocrine medical treatments for the therapeutic management of AgPitNETs/MetPitNETs due to the lack of randomized controlled clinical trials (Table 3). Therefore, with the exception of endocrine treatments prescribed for certain types of pituitary tumors, the majority of medical treatments used consist of off-label drugs, a significant portion of which are investigational drugs.

#### 3.3.1. Chemotherapy

Temozolomide (TMZ) is an oral active alkylating agent that acts through DNA methylation, causing irreversible DNA damage. Its effect can potentially be counteracted by the cellular repair protein O6-methylguanine-DNA methyltransferase (MGMT), a DNA repair protein that acts by removing methyl groups. TMZ is approved for the treatment of glioblastoma multiforme and has been used in a variety of solid tumors, including advanced NETs. Since 2006, TMZ has emerged as a therapeutic option for individuals with AgPitNETs/MetPitNETs refractory to conventional therapy with medical management and surgery with or without radiotherapy. Currently, TMZ is now considered a first-line treatment after failure of standard therapy in AgPitNETs/MetPitNETs as it has demonstrated the ability to improve 5-year progression-free and overall survival rates in responding patients [3,4,14,30,31]. 

However, many questions regarding the treatment of these tumors with TMZ remain to be clarified. These include the criteria for selecting appropriate patients for treatment, the knowledge of biomarkers predictive of a therapeutic response, the ideal therapeutic regimen, the appropriate timing of treatment initiation, the optimal duration of treatment, and the therapeutic options after treatment failure, such as the possibility of a second course of TMZ or combined therapy with other drugs and/or radiotherapy [30]. 

The recommended dose of TMZ as monotherapy is 150–200 mg/m^2^/day (repeated cycles of 5 days every 28 days). Evaluation of treatment efficacy after three cycles allows differentiation between responders and non-responders. If there is an adequate initial response, it is recommended that treatment be continued for a total of at least 6 months, with the potential for a longer duration if continued therapeutic benefit is observed [3]. 

Recently, the radiologic tumor response to TMZ has been documented as follows: CR in 9.6% of cases, PR in 30%, SD in 28%, and PD in 32% (Table 4). Notably, there were no significant differences in the response to TMZ between AgPitNETs and MetPitNETs. In addition, approximately one-third of patients with functioning tumors had a favorable hormonal response, characterized by a hormone reduction of more than 50%. The estimated mean time from the discontinuation of TMZ treatment to the initiation of subsequent treatment was 6.4, 3.3, and 1.4 years after achieving a CR, PR, and SD, respectively [9]. In a recent meta-analysis of 21 studies involving 429 patients, TMZ showed an objective response rate (CR + PR) of 41% with a hormonal response rate of 53% in functioning tumors. The 2- and 4-year survival rates were 79% and 61%, respectively. TMZ prolonged the median progression-free survival (PFS) and overall survival (OS) by 20.2 and 40.2 months, respectively [32]. 

TMZ is a generally well tolerated drug and does not require dose modification in patients with renal or hepatic insufficiency [34]. Approximately 20% of patients experience grades 2 to 4 adverse events associated with TMZ [32]. The most frequently reported adverse events are nausea, vomiting, constipation, anorexia, headache, fatigue, seizures, and rash. Prophylactic treatment with antiemetics (ondansertron) is recommended during the 5 days of the TMZ treatment cycle. It has been associated with myelotoxicity and hepatic involvement [14,30,31]. It is advisable to monitor each cycle for potential myelosuppression, as the most significant bone marrow suppression tends to occur around day 22 of the cycle [34]. Before starting a new cycle, it is advisable to ensure that the neutrophil and platelet counts are both above 1500 cells × mL and 100,000 platelets x mcl, respectively. In the event of myelosuppression, options include delaying TMZ treatment or reducing the TMZ dose [3].

While it appears that the assessment of MGMT status is critical in predicting a therapeutic response in other tumors (e.g., glioblastomas), it is still controversial in pituitary tumors. In a recent cohort study of 35 AgPitNET patients, all TMZ responders had <15% MGMT expression, whereas non-responders had an average of 50% MGMT expression [35]. A recent meta-analysis study showed that the radiologic response rate was significantly lower in patients with AgPitNET and MetPitNETs with high MGMT expression compared to that obtained in the patients with minimal and intermediate MGMT expression, with no significant difference between the latter two groups [32]. However, low MGMT expression does not guarantee a response to treatment. A review of aggressive TMZ-resistant prolactinomas showed that 35% had negative MGMT staining [36]. There is still concern about the inadequacies of MGMT analysis, including technical issues such as fixation methods, the need for and a lack of consensus on protocols for MGMT immunohistochemistry (IHC) evaluation, and the need for and a lack of consensus on protocols for MGMT IHC evaluation.

The optimal duration of TMZ treatment is currently unknown, although prolonged treatment appears to be associated with improved progression-free survival [31]. A recently published systematic review on the clinical follow-up of patients with AgPitNETs and MetPitNETs after discontinuation of TMZ showed considerable heterogeneity among the published papers. The duration of TMZ cycles ranged from 3 to 47 months; follow-up after TMZ discontinuation ranged from 4 to 91 months (mean 24 months, median 18 months). Furthermore, SD was reported in three-quarters of patients after a mean of 13 months (range 3 to 47 months, median 10 months) [37]. 

Currently, there is no established therapy available for patients who progress on TMZ. Potential therapeutic options include a second course of TMZ, immunotherapy, non-endocrine targeted therapies, and peptide receptor radionuclide therapy (PRRT) [31]. In cases of rapid tumor progression on TMZ, it is suggested to change the chemotherapy. If recurrence develops after an adequate initial response to TMZ, a new 3-month course of TMZ is suggested [3]. Approximately 20% of patients treated with TMZ receive a second course of therapy, with a median interval of 2.4 years for those who achieved a CR and 4.5 years for those who achieved a PR after the first course of treatment [9].

In patients with rapidly progressing tumors who have not received the maximum allowable doses of radiotherapy, combined treatment with TMZ and radiotherapy is a recommended approach [3]. When this combination therapy was used in patients with aggressive clinically functioning tumors, either according to the Stupp protocol (concurrent administration of TMZ at 75 mg/m^2^ per day and radiotherapy for 6 weeks, followed by TMZ alone at 150–200 mg/m^2^ per day, in 5-day cycles every 28 days for 6–12 months) [38] or when radiotherapy was administered within 6 weeks prior to discontinuation of TMZ, it resulted in a remarkable outcome, with 77.8% of patients achieving either a CR or PR [9]. There is currently an ongoing clinical trial assessing the benefits of the Stupp protocol compared to radiotherapy alone (ClinicalTrials.gov Identifier: NCT04244708) [39].

The combination of TMZ with other agents has also been described in a limited number of cases, most commonly with capecitabine (CAPTEM regimen) [40]. The potential benefit of this regimen has yielded mixed results, with no clear improvement in efficacy over TMZ monotherapy demonstrated to date. In addition, the additional toxicity that may develop must be considered [31]. A clinical study is currently underway to evaluate the efficacy and safety of a combined treatment approach with capecitabine and TMZ (ClinicalTrials.gov Identifier: NCT03930771) [41].

Other cytotoxic chemotherapeutic agents have been used in a limited number of patients with AgPitNETs/MetPitNETs, most notably lomustine as monotherapy or in combination with 5-FU [42]. In addition, adriamycin, cisplatin, carboplatin, and others have been studied in various combinations. However, the results show rather modest efficacy with more adverse side effect profiles, although in some patients the disease has stabilized and a PR has even been achieved [9].

#### 3.3.2. Pituitary-Targeted Therapies 

Approved therapies for the treatment of functioning pituitary tumors include dopamine agonists (DAs) for prolactinoma and first- and second-generation somatostatin analogs (SSAs) for the treatment of acromegaly and Cushing’s disease. In cases of functioning AgPitNETs/MetPitNETs, treatment with these pharmacological agents at the maximum tolerated dose is recommended to control tumor growth and hormone hypersecretion. In some cases of aggressive tumors, treatment with off-label pituitary therapies may be considered. However, their use is supported only by isolated clinical cases or small published case series.

##### Dopamine Agonists

Dopamine type 2 receptors (D2Rs) have been reported to be expressed in approximately 80% of functioning corticotropinomas [43]. In this context, normalization of cortisol secretion has been described in up to 20–40% of patients with Cushing’s disease treated with cabergoline [43,44]. However, treatment escape of DAs has been reported in 18–33% of patients with Cushing’s disease [45]. The role of DAs in corticotropin secretion and tumor volume in aggressive corticotropinomas is not really known [46,47]. Therapeutic experience with cabergoline in Nelson’s syndrome is limited [48], although there have been some isolated clinical cases in which it was effective [49,50,51]. In 2001, the first case of a tumor response to cabergoline was reported in a silent corticotropinoma [52]. In vitro study of the tumor showed the presence of D2 receptors at levels similar to those found in control prolactinomas. These findings suggest that a therapeutic trial with cabergoline should be considered in cases of recurrent silent corticotropinoma.

DAs have found their therapeutic place in patients with non-aggressive acromegaly and low secretory activity [53]. Cabergoline has been shown to induce potential tumor shrinkage. This is particularly true in patients with acromegaly who have mixed GH- and prolactin-secreting adenomas [54]. However, these drugs have been reported to improve the response rate to SSA, and combination therapy with SSA and pegvisomant may be an option for aggressive, non-responsive tumors in patients with acromegaly. Several studies have shown a 30–40% normalization of IGF-1 and a 12.5 mm^3^ reduction in tumor volume in octreotide-resistant patients treated with cabergoline and octreotide [55,56]. The potential therapeutic role of DAs in aggressive clinically functional or silent GH-secreting PitNETs is currently unknown.

D2R expression and function have been demonstrated in nearly 70% of clinically non-functioning pituitary tumors. This suggests a role for this drug in the treatment regimen of these tumors [57]. Cabergoline appears to be an effective drug for the treatment of postoperative remnants of NFPAs, as it has been associated with a significant rate (13.6–38%) of tumor shrinkage [58,59,60]. Although the therapeutic role of DAs in AgPitNETs is not fully known, it is possible that they may play a beneficial role in certain cases when used as part of a combined therapeutic approach.

##### Somatostatin Analogs

Prolactinomas that do not respond to DAs are uncommon and may also exhibit aggressive or even malignant behavior. They represent a significant challenge due to the limited treatment options available for patients with this condition [61]. PRL-secreting tumors often show the presence of somatostatin receptors (SSTRs), with notable expression of SSTR5 and SSTR1, and lesser expression of SSTR2 [62,63]. Isolated clinical cases [64] and small series of patients [65] with DA-resistant maroprolactinoma have been reported showing that the addition of a first-generation somatostatin analog (octreotide LAR) to ongoing cabergoline treatment may be effective in these patients, regardless of the SSTR expression profile of the adenoma [65].

In recent years, numerous studies have shown that SSTRs are commonly expressed in non-functioning pituitary adenomas (NFPAs), mainly the SSTR5 and SSTR3 subtypes [66]. It has also been reported that long-term treatment with octreotide LAR correlates with stabilization of residual tumors after surgery for NFPAs in 81% of cases, with only a modest effect on tumor size reduction [67]. The potential therapeutic role of SSAs in aggressive NFPAs is currently unknown.

Pasireotide, a novel multireceptor-targeted SSTR ligand (SRL) with higher affinity to SSTR 5 than SSTR2, unlike first-generation SRLs, has been introduced as a potential alternative for the treatment of aggressive or DA-resistant prolactin PitNETs in isolated case reports [68,69,70]. In one case, co-treatment with pasireotide LAR and cabergoline resulted in PRL normalization within two months that was maintained for 31 months and a PR during 23 months of treatment [68].

Similarly, isolated clinical cases have been documented in which pasireotide has been associated with a decrease in both plasma ACTH levels and tumor volume among patients diagnosed with Nelson’s syndrome [71,72,73]. One patient with Nelson’s syndrome showed and early response to pasireotide with a 90% reduction in plasma ACTH and SD levels after 2 months of treatment [72].

#### 3.3.3. Non-Endocrine Targeted Therapies

##### Anti-Vascular Endothelial Growth Factor Therapy

Increased vascular density and vascular endothelial growth factor (VEGF) expression have been described in aggressive pituitary tumors [74,75]. VEGF is a dimeric glycoprotein that acts as an angiogenic cytokine that stimulates cell division. Therefore, the use of drugs that inhibit VEGF function may prevent the formation of new blood vessels that tumors need to grow. Bevacizumab (BVZ), a humanized monoclonal antibody against VEGF, is one of the first targeted cancer therapies and the first approved anti-angiogenic agent [76]. In an analysis of 17 patients (10 AgPitNETs and 7 MetNETs; 6 corticotrophs, 2 lactotrophs, 1 somatotroph, and 8 of unknown histology) treated with BVZ, of which 15 were previously treated with TMZ and 7 received combined treatment (TMZ + BVZ), the radiologic response evaluated in 15 of them showed a CR in 1 patient, PR in 4, SD in 7, and PD in 3 [15] (Table 4). These results show that tumor progression was prevented in 80% of patients, with an objective response rate (CR plus PR) of 34%, suggesting that BVZ could be tried after failure of TMZ [10]. Although anti-VEGF therapy shows promising results in the treatment of aggressive pituitary tumors, its use remains controversial due to the lack of large-scale clinical trials. Therefore, further preclinical research and clinical trials are needed to more comprehensively evaluate the efficacy and safety of anti-VEGF therapies in AgPitNETs/MetPitNETs [11].

##### Tyrosine Kinase Inhibitors

Tyrosine kinase inhibitors (TKIs) are a form of targeted therapy that can block the activity of TKs. These enzymes are responsible for triggering the activation of various proteins through signal transduction pathways, which contribute to the control of various aspects of cell function, such as signaling, cell growth, and proliferation [77]. Accordingly, TKIs prevent cancer cell growth by inhibiting the TK activity of various growth factor receptors (GFRs) [78,79]. At present, the factors responsible for the pathogenic processes of pituitary tumor initiation, expansion, and invasion are currently unclear. Recent studies suggest that neurotrophins and other GFs play an important role in the initiation and spread of pituitary adenomas [80]. Among these is epidermal growth factor (EGF), which is characterized by its mitogenic activity and is thus involved in the process of cell growth and tumorigenesis through binding to the EGF receptor (EGF-R), a 170 kDa transmembrane glycoprotein with TK activity. Various studies have reported a moderate to high percentage of EGFR overexpression in pituitary adenomas, preferentially in non-functioning versus functioning adenomas [81,82,83]. Therefore, the use of EGFR-targeted inhibitors would be an attractive therapeutic alternative in the treatment of aggressive pituitary tumors, given their proven efficacy in other cancers [84]. To date, TKIs have only been used to treat a small number of patients with aggressive pituitary adenomas/carcinomas. In an analysis of 12 patients (10 AgPitNETs and 2 MetPitNETs), they were treated with lapatinib (*n* = 8), sunitinib (*n* = 2), erlotinib (*n* = 1), or apatinib (*n* = 1). The radiologic response was PR in one patient, SD in five patients, and PD in six patients [15].

##### PI3K/AKT/mTOR Signaling Pathway Inhibitors

The PI3K/AKT/mTOR signaling pathway is activated by various stimuli such as growth factors, nutrients, energy, and stress signals to control cell growth, proliferation, and survival. Dysregulation of several elements of the mTOR signaling pathway has been described in various cancers [85]. The PI3K/AKT/mTOR pathway has been shown to be overexpressed in both hormonally active and inactive pituitary adenomas compared to normal pituitary, and has also been reported to be upregulated in invasive pituitary tumors [86,87]. Therefore, mTOR is an attractive therapeutic target and mTOR inhibitors such as rapamycin and everolimus may be of therapeutic interest in AgPitNETs/Met/PitNETs [88]. Although some in vitro studies have shown that EVE induces apoptosis in non-functioning pituitary tumors [89], these results have not been reproduced in patients with aggressive tumors. To date, everolimus (EVE) is the only PI3K/AKT/mTOR pathway inhibitor that has been studied in patients with aggressive pituitary tumors, with a total of seven documented cases (four AgPitNETS and three MetPitNET; three corticotrophs, one lactotroph and three of unknown histology) [12,90,91,92]. Of these, only the patient with aggressive prolactinoma had a biochemical response with reduced prolactin levels and a radiographic response as SD for 12 months [90], while the rest showed PD.

Although the PI3K/AKT/mTOR signaling pathway plays critical roles in the regulation of tumor cell metabolism, growth, and survival, there is a need to investigate the underlying mechanisms of these pathways and their possible relationship with the pathogenesis, invasiveness, aggressive behavior, and refractoriness to conventional treatment of PitNETs [88].

### 3.4. Hormonal Antisecretory Drugs

In aggressive hyperfunctioning pituitary tumors, the use of hormone antisecretory drugs is critical to control hormone overproduction in the peripheral endocrine glands. For example, in Cushing’s disease, adrenal steroidogenesis inhibitors such as ketoconazole, metopirone, or osilodrostat, and cortisol receptor antagonists such as mifepristone are essential to regulate excessive adrenal cortisol secretion or to reduce its effects [93]. Similarly, in acromegaly, GH receptor antagonists such as pegvisomant have a key role in controlling hepatic overproduction of IGF-1 [94]. In these patients, it is advisable to use the highest tolerated dose of antisecretory drugs to effectively control excessive hormone secretion.

Although a slightly increased risk of tumor growth has been reported with the use of monotherapy anti-hormonal secretion drugs such as pegvisomant in patients with acromegaly [95], multimodal treatment with surgery, radiotherapy, and combined medical treatment (drugs directed against pituitary tumor + hormonal antisecretory drugs) used in aggressive GH-secreting tumors reduces this possibility.

### 3.5. Immunotherapy

Immunotherapy is a treatment that uses the body’s own immune system to treat various diseases, particularly cancer and autoimmune diseases [96,97]. There are several types of immunotherapy, each of which is used differently depending on the type of disease and the needs of the patient. These include immune checkpoint inhibitors (ICIs) [98]. These are antibodies that block immune checkpoint proteins (CTLA-4 and PD-1/PD-L1), molecules that help cancer cells prevent the immune system from attacking them [99,100]. CTLA-4 blocking (anti-CTLA-4) ICIs, such as ipilimumab and tremelimumab, PD-1 blocking (anti-PD-1) ICIs, such as nivolumab and pembrolizumab, and PD-L1 blocking (anti-PD-L1) ICIs, such as atezolizumab, durvalumab, and avelumab, help T cells stay active and attack cancer cells. ICIs are effective in the treatment of several cancers, including melanoma and non-small cell lung cancer, where they have been shown to improve survival and quality of life in patients [101].

Some studies have shown that both functioning PitNETs and AgPitNETs contain infiltrating lymphocytes and express PD-L1 [102,103]. Upregulation of PD-L1 may represent a pathway to evade immune surveillance by suppressing the host immune response and thus may be associated with more aggressive behavior in PitNET [104]. These results would indicate the existence of an immune response against some types of aggressive pituitary tumors and would raise the possibility of the therapeutic use of ICIs in cases refractory to conventional treatment, such as AgPitNETs/MetPitNETs. The efficacy of ICI therapy (anti-PD-L1) in a mouse model of Cushing’s disease has been recently reported [105]. To date, only 24 cases have been treated with ICIs: 16 corticotroph (9 MetPitNETs and 7 AgPitNETs) and 8 lactotroph (4 AgPitNETs and 4 MetPitNETs) tumors. All of them had previously received multimodal treatment, including TMZ [33]. Their radiologic responses are shown in Table 4.

TMZ has the potential to induce changes in the mismatch repair system. As a result, this may lead to increased production of neoantigens, which in turn could increase the efficacy of ICI immunotherapy [106]. In fact, the development of somatic hypermutation caused by conventional chemotherapy (CAPTEM) has been reported to be associated with a better response to ICI therapy in a patient with an ACTH-secreting MetPitNET [107]. 

Although combination therapy with ipilimumab and nivolumab could be an effective therapeutic option for patients with MetPitNETs, no clear advantage over monotherapy has been documented to date. The first case of a prolactin-secreting MetPitNET went into remission that was maintained for 24 months after treatment with ICI (nivolumab and ipilimumab initially, followed by maintenance treatment with nivolumab every 2 weeks) was recently reported [108]. Finally, negative immunostaining for PD-L1 does not completely rule out the possibility of a therapeutic response to immunotherapy.

Two ongoing clinical trials, registered under the identifiers NCT04042753 and NCT02834013, are currently evaluating the impact of combined therapy with nivolumab and ipilimumab in patients with AgPitNETs/MetPitNETS [109,110].

### 3.6. Peptide Receptor Radionuclide Therapy 

The presence of SSTRs on the surface of the tumor cell is the molecular basis for both diagnosis and treatment (known as “teragnosis”) of NETs. The expression of SSTRs in most subtypes of PitNETs [62,63,66] raises the possibility of a therapeutic role for radiolabeled SSAs in these tumors. The expression of SSTR2 in the tumor can be detected by SPECT/CT scan with octreotide (Octreoscan, Tektrotyd^®^) or by positron emission tomography (PET)/CT with 68Ga-DOTA-TATE. Although 68Ga-DOTA-TATE/TOC uptake in PitNETs is high, it is also elevated in the normal pituitary due to high expression of SSTR2. However, there is no threshold SUVmax (maximum standardized uptake value) to definitively differentiate between normal pituitary and PitNETs. Peptide receptor radionuclide therapy (PRRT) with radiolabeled SSAs has been shown to be an effective treatment in patients with well to moderately differentiated metastatic NETs expressing SSRs [111]. PRRT therapy, using compounds such as 90Y-DOTA-TOC/TATE and 177Lu-DOTA-TATE/TOC, is emerging as a promising therapeutic option for patients with AgPitNETs/MetPitNETs with high radiolabeled SSA uptake. To date, there is limited experience with PRRT in these patients, with therapeutic outcomes reported as isolated clinical cases or small series of patients [5,9,112,113,114,115,116,117,118].

In the recent ESE study by Burman et al. [9] performed in 171 patients with aggressive pituitary tumors (121 AgPitNET and 50 MetPitNET), 11 patients (6.4%) (10 AgPitNET, including 5 prolactinomas, 4 non-functioning tumors, and 1 TSHoma; and 1 GH-secreting MetPitNET) were treated with PRRT (8 with 177Lu-DOTA-TATE/TOC, 3 with 90Yttrium-DOTA-TOC, and one with 111In-DTPA-octreotide). One of them was managed with 90Yttrium-DOTA-TOC (×2 cycles) and 177Lu-DOTA-TATE (×1 cycle). A PR and SD were achieved in three and two patients, respectively. Of the two patients who achieved SD, one patient maintained SD for at least 12 months and the other patient maintained adequate tumor control for 15 years (Table 4). Hematologic cytopenias, increased facial pain, and pituitary apoplexy have been described as adverse effects associated with PRRT [15].

New clinical research strategies have also been proposed to improve the outcomes of PRRT in patients with AgPitNETs. These strategies include its early use and the utilization of new radioligands, such as radiolabeled somatostatin antagonists instead of SSA or radioligands with different affinity profiles for SSTR subtypes. In addition, the possibility of combining PRRT with radiosensitizers, i.e., drugs capable of increasing the expression of SSTRs, or with immunotherapy, has been raised [119,120,121].

## 4. Conclusions

**Figure 2:**
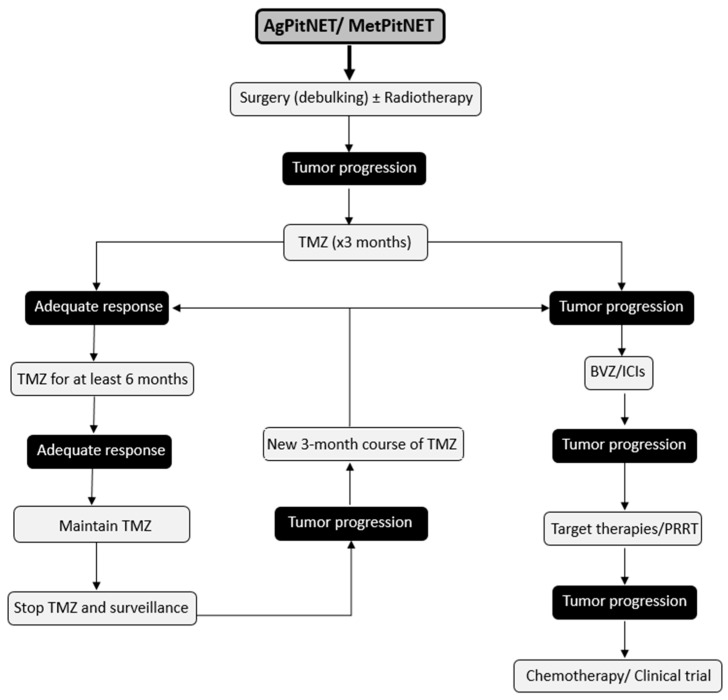
Suggested Therapeutic Management for Patients with AgPitNET/MetPitNETs.

AgPitNET/MetPitNET are extremely rare pituitary tumors and their treatment is a major clinical challenge due to their aggressive clinical behavior. Early detection of these tumors is critical due to their aggressive behavior and high morbidity and mortality. This allows for an aggressive therapeutic approach from the time of detection. After failure of conventional approaches, which include medical management, surgery, and radiotherapy, reintervention for tumor debulking surgery should be considered whenever feasible. In addition, a new course of radiotherapy and initiation of chemotherapy with TMZ should be considered as first-line treatment, as it has been shown to increase the 5-year progression-free survival rate and overall survival. A positive initial response to TMZ suggests the feasibility of a second cycle in the event of tumor progression. When treatment with TMZ is unsuccessful, other therapeutic options can be explored, such as BVZ, ICIs, PRRT, and targeted therapies (Figure 2). It is important to consider the publication bias in reports of new treatment options and to recognize how this factor is a limitation of the available literature.

Currently, there are no clinical guidelines that clearly define the ideal therapeutic strategy based on the response to different treatments used in the management of these tumors. This lack of guidance is partly due to the absence of comparative clinical trials evaluating different therapeutic options. Therefore, the proposed therapeutic strategy is based on the results from retrospective studies and the clinical responses observed in the small patient series that have been published to date. It should not be considered as a rigid approach but rather as a recommendation that may vary from patient to patient. Additionally, this strategy may evolve in the future based on new studies that are published.

Future perspectives should be aimed at improving the precision of surgical procedures, using advanced radiotherapy techniques, exploring new combinations of drugs or pharmacological treatment in combination with radiotherapy, and incorporating therapies directed against specific molecules that stimulate tumor progression. In addition, it is essential to identify new, more precise predictive markers to facilitate the early detection of these tumors, which would allow rapid intervention and avoid complications associated with multiple recurrences and/or distant metastases. The response of these tumors to the many and varied potential therapeutic alternatives should be evaluated. The overall goal of this research is therefore to develop treatments that are not only more effective, but also better tolerated, in order to offer the best options to patients with AgPitNET/MetPitNETS.

## Figures and Tables

**Table 1:** Main clinical and pathological criteria for considering the diagnosis of an aggressive pituitary neuroendocrine tumor (AgPitNET). The presence of at least 3 of the following criteria would be required to establish the diagnosis.

1. Rapidly growing and/or large tumor size
2. Invasive tumor (≥1 criterion) Cavernous sinus or sphenoid sinus Bone Nasal mucosa
3. High cell proliferation (≥2 criteria) Ki-67 ≥ 3%. Mitoses > 2 per 10 HPFs p53 (>10 nuclei per 10 HPFs)
4. Refractory to standard treatment (medical, surgery, and/or radiotherapy)
5. Recurrence/progression

Abbreviation: HPF, high power field. Adapted from [3].

**Table 2:** Main histological subtypes of PitNETs that usually show aggressive behavior.

1. Silent corticotropinoma
2. Densely granular lactotroph adenoma (males)
3. Sparsely granulated somatotropic adenoma
4. Crooke’s cell adenoma
5. Immature plurihormonal tumor of PIT1 lineage
6. Acidophilic stem cell adenoma
7. Null cell tumor

**Table 3:** Approved and off-label drugs used in the pharmacological management of pituitary tumors.

	Approved	Off-Label
Dopamine agonists *Bromocriptine* *Cabergoline*	Prolactinoma	Acromegaly Cushing’s disease Nelson’s syndrome NFPitNET
First-generation SSA *Octreotide* *Lanreotide*	Acromegaly Thyrotropinoma GEPNETs	Prolactinoma NFPitNET
Second-generation SSA *Pasireotide*	Acromegaly Cushing’s disease	Aggressive prolactinoma Silent corticotropinoma Nelson’s syndrome
*TMZ*	Glioblastoma multiforme	AgPitNET MetPitNET

Abbreviations: AgPitNET, Aggressive pituitary neuroendocrine tumor; GEP-NET, Gastroenteropancreatic neuroendocrine tumor; MetPitNET, Metastatic pituitary neuroendocrine tumor; NFPitNET, Non-functioning pituitary neuroendocrine tumor; SSA: Somatostatin analog; TMZ, Temozolomide.

**Table 4:** Tumor radiological response to different therapies in patients with AgPitNETs and MetPitNETs.

	CR	PR	SD	PD
Temozolomide, *n* = 156 (%)	9.6	30.1	28.1	32.2
Bevacizumab, *n* = 15 (%)	6.7	26.7	46.7	20.0
Immunotherapy (ICI), *n* = 24				
Corticotroph, *n* = 16 (%)	6.25	37.5	12.5	43.7
Lactotroph *n* = 8 (%)	0	25.0	12.5	62.5
PPRT, *n* = 11 (%)	0	27.3	18.2	54.5

Abbreviations: CR, complete response; ICI, Immune checkpoint inhibitors; PD, progressive disease; PPRT, Peptide receptor radionuclide therapy; PR, partial response; SD, stable disease. Adapted from [9,15,33].

## Data Availability

Data sharing is not applicable to this article.
